# QSAR Study of *N*-Myristoyltransferase Inhibitors of Antimalarial Agents

**DOI:** 10.3390/molecules23092348

**Published:** 2018-09-13

**Authors:** Letícia Santos-Garcia, Marco Antônio de Mecenas Filho, Kamil Musilek, Kamil Kuca, Teodorico Castro Ramalho, Elaine Fontes Ferreira da Cunha

**Affiliations:** 1 Departamento de Química, Universidade Federal de Lavras, Lavras 37200-000, Brazil; leticiasantosg@hotmail.com (L.S.-G.); marcomecenasfilho@gmail.com (M.A.d.M.F.); teo@dqi.ufla.br (T.C.R.); 2 Department of Chemistry, Faculty of Science, University of Hradec Kralove, Hradec Kralove 50005, Czech Republic; kamil.musilek@uhk.cz; 3 Biomedical Research Center, University Hospital Hradec Kralove, Hradec Kralove 50005, Czech Republic; 4 Center for Basic and Applied Research, University Hradec Kralove, Hradec Kralove 50005, Czech Republic

**Keywords:** malaria, *N*-myristoyltransferase, drug development, QSAR, mosquito-borne protozoal infection

## Abstract

Malaria is a disease caused by protozoan parasites of the genus *Plasmodium* that affects millions of people worldwide. In recent years there have been parasite resistances to several drugs, including the first-line antimalarial treatment. With the aim of proposing new drugs candidates for the treatment of disease, Quantitative Structure–Activity Relationship (QSAR) methodology was applied to 83 *N*-myristoyltransferase inhibitors, synthesized by Leatherbarrow et al. The QSAR models were developed using 63 compounds, the training set, and externally validated using 20 compounds, the test set. Ten different alignments for the two test sets were tested and the models were generated by the technique that combines genetic algorithms and partial least squares. The best model shows r^2^ = 0.757, q^2^_adjusted_ = 0.634, R^2^_pred_ = 0.746, R^2^_m_ = 0.716, ∆R^2^_m_ = 0.133, R^2^_p_ = 0.609, and R^2^_r_ = 0.110. This work suggested a good correlation with the experimental results and allows the design of new potent *N*-myristoyltransferase inhibitors.

## 1. Introduction

**Figure 1:**
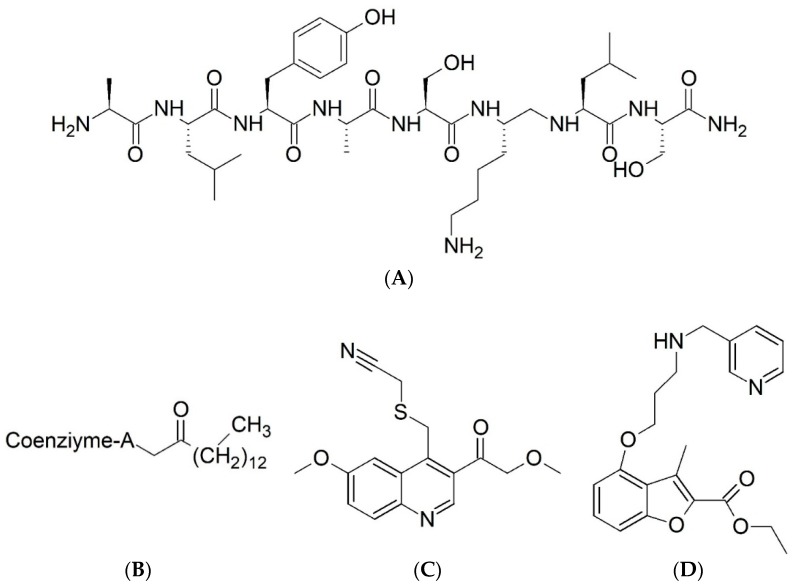
(**A**) Inhibitor mimicking the structure of substrates (*Ki* = 5.0, 8.0, and 35.0 μM for *S. cerevisiae*, *C. albicans*, and human NMT) [12]; (**B**) inhibitor methylene-bridged analogue of myristoyl coenzyme A (*Ki* = 24.0 nM) [13]; (**C**) inhibitor based on a quinolone scaffold (*Ki* = 4.7, and >100 μM for *Plasmodium vivax* and *Plasmodium falciparum NMT*, respectively); and (**D**) inhibitor based on a furan core RO-09-4609 (IC_50_ = 0.1 and >540 μM for *C. albicans*, and human NMT) [14].

Malaria is a mosquito-borne protozoal infection caused by five parasites of the genus *Plasmodium*: *P. falciparum*, *P. ovale*, *P. malariae*, *P. vivax*, and *P. knowlesi* [1]. Among these, *Plasmodium falciparum* is the most prevalent and lethal [2]. Over the past 50 years, the parasite resistance to chloroquine and sulphadoxine-pyrimethamine in endemic countries has been noted [3,4]. In addition, signs of resistance to artemisinin-based combination therapies (ACTs) have been detected. Actually, ACTs are the first-line treatment for malaria and thus, new drugs are constantly required [5,6,7,8]. Researchers have identified the *N*-myristoyltransferase (NMT) enzyme as an important target for a generation of drugs to be used for the treatment of malaria. NMT catalyzes the transfer of the myristoyl group from a myristoyl coenzyme A (CoA) to the N-terminal glycine residue after the targeted protein [9]. N-terminal myristoylation (MYR) by NMT occurs through the Bi-Bi mechanism, where MYR-CoA binds to the apo-enzyme, inducing a conformational change that allows the NMT substrate to bind [5,10]. *P. falciparum* has a single NMT isoform and mRNA is expressed in asexual blood-stage forms [11]. The first reported NMT inhibitors were obtained by mimicking the structure of peptide substrates (Figure 1A) [12] or by designing non hydrolysable, methylene-bridged analogue of myristoyl coenzyme A (Figure 1B) [13]. After that, inhibitors based on a quinolone scaffold and furan core were reported (Figure 1C,D) [14]. In this work, we used dimensional quantitative structure-activity relationship analysis of 83 NMT inhibitors based on a phenyl scaffold [15] seeking to propose new candidates for NMT inhibitors. Furthermore, a physicochemical properties evaluation was performed in order to find the most appropriate compound predicted.

## 2. Results

The GA-PLS analysis using grid cells of 1.0 A generated several models or equations. The statistical parameters of ten alignments studied for Test Set I (compounds **1**, **3**, **5**, **6**, **12**, **16**, **20**, **30**, **33**, **39**, **40**, **50**, **56**, **57**, **61**, **65**, **66**, **69**, **76**, and **80**) and Test Set II (compounds **3**, **6**, **9**, **13**, **20**, **21**, **27**, **28**, **31**, **32**, **40**, **56**, **57**, **58**, **64**, **70**, **73**, **76**, **78**, and **82**) are shown in Table 1 and Table 2, respectively. All tested alignments showed q^2^ values higher than 0.5. This reveals that the model can be a useful tool for predicting affinities of new compounds based on these structures; r^2^ greater than 0.7 indicates that the model is correlated and may be considered to represent the training set in the same manner [16]. Alignments 6B and 7B were eliminated from the analysis because it presented a low r^2^ value (<0.7).

Evaluating the predictive ability in terms of R^2^_pred_ (means of an external validation), the alignment 5A was eliminated (R^2^_pred_ < 0.5). All R^2^_m_ values were greater than 0.60, and values over 0.5 are acceptable. Analyzing the R^2^_p_ values, alignments B1, B2, B4, B6, and B7 were excluded because this parameter value was less than 0.5 [17].

Alignment 3 from Test Set II (B3) provides the best 4D-QSAR models as judged by the highest q^2^_adj_, in addition to presenting fewer descriptors. Among the alignments with only seven descriptors, this still has the highest values of r^2^, q^2^_adj_, R^2^_p_, and the lowest value of R^2^_r_. According to these results, we selected Model B3 as the best alignment. We will only present the analysis of the best model derived from B3.

The statistical measures, including the values of r^2^, q^2^, q^2^_adj_, LSE, LOF, RMSE_C_, RMSE_CV_, RMSE_P_, Y-Rand, R^2^_pred_, R^2^_m_, R^2^_p_, and R^2^_r_ are presented below. Each GCOD (grid cell occupancy descriptors) is labeled as “x, y, z, IPE” which represent the cartesian coordinate positions of the selected grid cell (x, y, z) and the respective atom type (interaction pharmacophore elements, IPE): (i) any type (any); (ii) nonpolar (np); (iii) polar-positive charge density (p+); (iv) polar-negative charge density (p−); (v) hydrogen bond acceptor (hba); (vi) hydrogen bond donor (hbd); and (vii) aromatic systems (ar).

### Model B3

pIC_50_ = 3.997 + 4.942(0,−3,−1, hba) + 2.345(0,−5,−1, any) + 2.100(0,−1,0, any) + 1.692(0,3,−3, any) + 1.191(−1,−4,−3, any) ‒ 8.269(−1,−4,−4, np)

*n* = 63, GCODs = 7, r^2^ = 0.757, q^2^ = 0.702, q^2^_adj_ = 0.634, LSE = 0.233, LOF = 0.418, RMSE_C_ = 0.472, RMSE_CV_ = 0.527, RMSE_P_ = 0.515, RMSE_cy-rand_ = 1.055, R^2^_pred_ = 0.746, R^2^_m_ = 0.716, R^2^_p_ = 0.609, and R^2^_r_ = 0.110.

Another different variant of R^2^_m_ metrics was calculated from Model B3 to assess the predictive ability of the test set, ∆R^2^_m_. The value of ∆R^2^_m_ found was 0.133. It has been suggested that to be considered a predictive model, this value should be less than 0.2 [18]. Model B3 generated seven descriptors, where GCODs (−1,−4,−3, any), (0−0, any), (0,6,2, any), (0,−5,−1, any), (0,3,−3, any), and (0,−3, −1, hba) present positive coefficients (Equation (3)) and correspond to favorable interactions between the molecule substituent and amino acid residues in the active site of NMT. Therefore, substituents in these positions increase the effectiveness of the compounds. The GCOD (−1,−4,−4, np) has negative coefficient and correspond to unfavorable interactions between the molecule substituent and amino acid residues in the active site of NMT. Therefore, the occupation of GCOD (−1,−4,−4, np) decreases the compound potency.

## 3. Discussion

**Figure 2:**
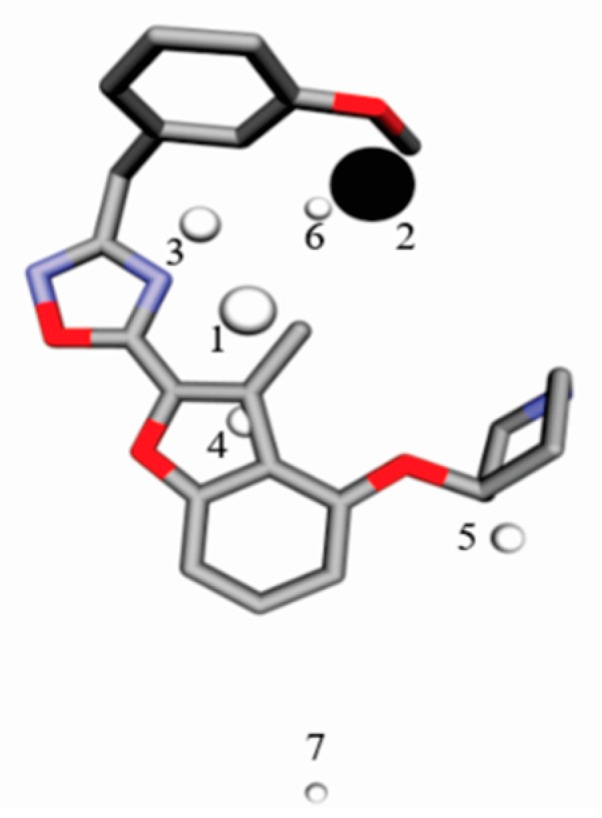
Graphic representation of Compound **81** according to the 4D-QSAR Model B3. GCODs occupancy represented by white spheres contributes to increasing the potency of compounds, and black spheres to decrease the potency of the compounds. The GCODs described are: (1) (0,−3,−1, hba), (2) (−1,−4,−4, np), (3) (0,−5,−1, any) (4) (0,−1,0, any), (5) (0,3,−3, any) (6) (−1,−4,−3, any), and (7) (0,6,2, any). The gray and red representations are carbons and oxygen atoms.

**Figure 3:**
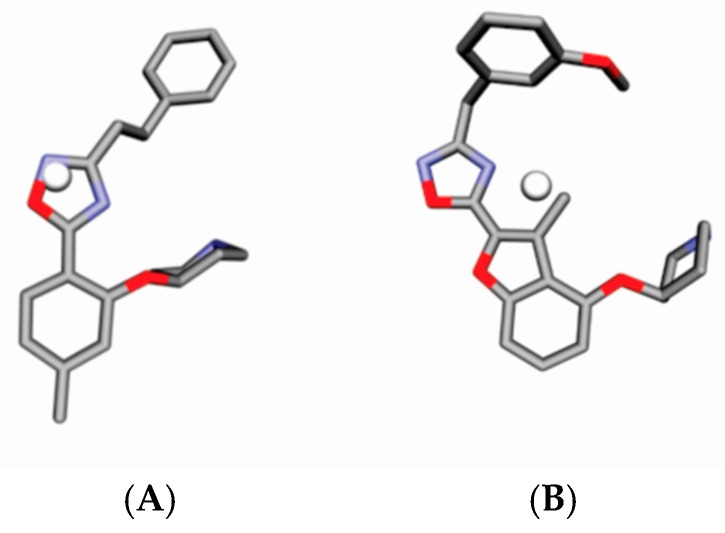
(**A**) Representation of compound **42** and GCOD-1 (0,−3,−1, hba) (white sphere), and (**B**) compound **81**. GCODs occupancy represented by white spheres contributes to increasing the potency of compounds. The gray and red representations are carbons and oxygen atoms.

GCODs are related to the coordinates of IPE mapped in a common grid. A graphic representation of the descriptors of Model B3 is shown in Figure 2 using Compound **81** as a reference. Light and dark spheres represent GCODs with positive and negative coefficients, respectively, in accordance with Model B3. GCOD-1 (0,−3,−1, hba) (Figure 3) is the descriptor that most contributes to the increased effectiveness of compounds and presents a coefficient of 4.942. This grid cell represents an acceptor hydrogen bond atom type (IPE) and shows high frequency of occupation for compounds **42**, **48**, **65**, **68**, and **69**. It is located close to the nitrogen atom of the oxadiazole ring and indicates an amino acid donor hydrogen bond in *N*-myristoyltransferase.

The oxadiazole ring in the ortho position allows the nitrogen atom to occupy this grid cell, exemplified by compound **42** (Figure 3). However, the most active molecules of the training set, **81** and **83**, do not have this descriptor that contributes most to the increase in the potency of the compounds. Once in this position there is a methyl group and the oxadiazole group that are displaced. In fact, the oxadiazole ring in these compounds does not occupy this grid cell.

**Figure 4:**
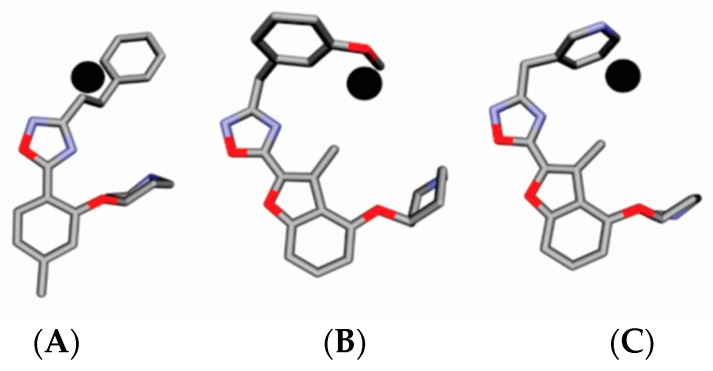
Representation of GCOD-2 (−1,−4,−4, np) (black sphere) and (**A**) compound **42**, (**B**) compound **81**, and (**C**) compound **83**. CODs occupancy represented by black spheres to decrease the potency of the compounds. The gray and red representations are carbons and oxygen atoms.

GCOD-2 (−1,−4,−4, np) (Figure 4) contributes to decrease compound potency and presents a coefficient of −8.269. This grid cell corresponds to a nonpolar IPE and shows high occupation frequency for Compounds **42**, **48**, and **55**. These molecules present non-polar groups in this local, such as ethyl and methyl groups. Thus, the occupation of this cell is drastically reduced when this position is non-polar substituted, that decrease the activity of these compounds. Meanwhile, if the polar groups, such as OH or N in **81** or **83**, respectively, are localized in this grid cell, the GCOD-2 descriptor yields less negative value which does not decrease the predicted pIC_50_. We can see that compound **42** occupies the descriptor that decreases the activity. However, it also presents occupancy for GCOD-1, which increases the activity. As expected, this compound exhibits an intermediate power.

This suggests that the occupation of this cell by acceptor hydrogen bond should be favorable in contrast to nonpolar atoms that are unable to perform hydrogen bond interactions.

**Figure 5:**
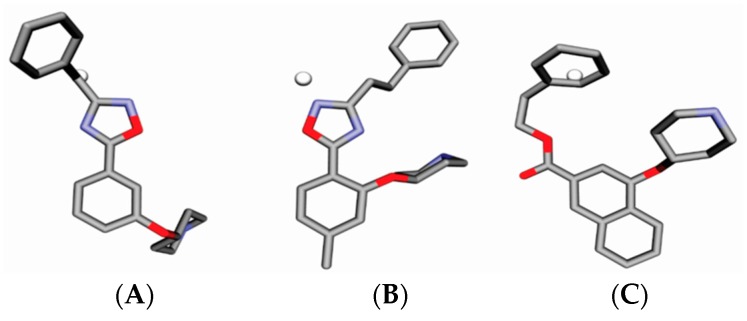
Representation of GCOD-3 (0,−5,−1, any) (white sphere) and (**A**) compound **30**, (**B**) compound **42**, and (**C**) compound **60**. CODs occupancy represented by white spheres contributes to increasing the potency of compounds. The gray and red representations are carbons and oxygen atoms.

GCOD-3 (0,−5,−1, any) is present as a non-specific IPE. The atom type “any” is used when more than one specific atom [IPE] type across the training set is found to satisfy the interaction being captured by a particular GCOD [19]. GCOD (0,−5,−1, any) has a positive coefficient of 2.345 which increases the potency of the compounds (Figure 5). This grid cell is close to methyl and ethyl groups, toward the left side, and shows greater occupation frequency for compounds **26**, **30**, and **33**. On the other hand, compounds **42** and **45** showed no occupancy for this descriptor, because during the molecular dynamics simulation, they assumed a different conformation, facing right. On the other hand, GCOD (0,−5,−1, any) also provides occupation frequency in the benzene ring of molecules **59**, **60**, **61**, and **62**.

**Figure 6:**
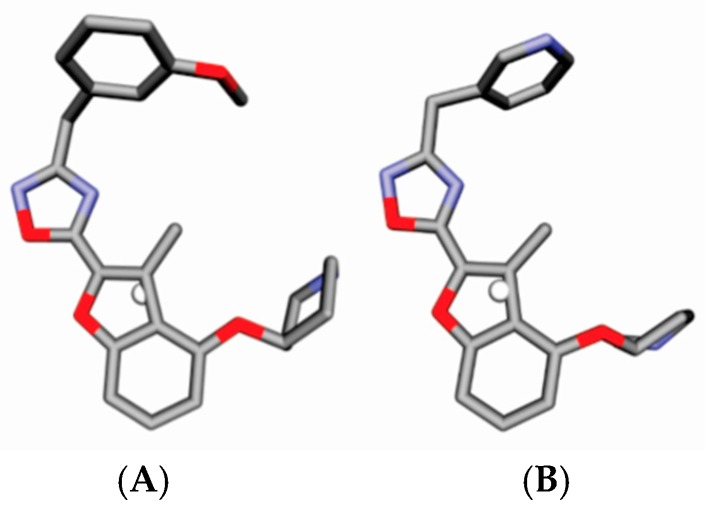
Representation of GCOD-4 (0,−1,0, any) (white sphere) and (**A**) compound **81**, and (**B**) compound **83**. CODs occupancy represented by white spheres contributes to increasing the potency of compounds, and the gray and red representations are carbons and oxygen atoms.

GCOD-4 (0,−1,0, any) have a positive coefficient and, thus, also greatly influence the increase in inhibitor potency (Figure 6). It is located near the methyl group in benzofuran, 2,3-dihidro-3-methyl and represents a non-specific IPE. It shows the highest occupation frequency for the most active compounds in this series, compounds **1**, **81**, and **83**. The GCOD (0,−3,−1, hba) reflects the importance of occupation of this receptor region for the effectiveness of the inhibitors.

**Figure 7:**
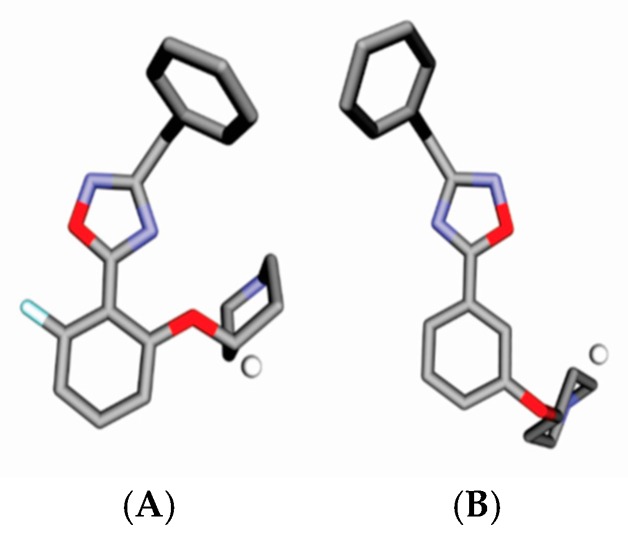
Representation of GCOD-5 (0,3,−3, any) (white sphere) and (**A**) compound **79**, and (**B**) compound **30**. CODs occupancy represented by white spheres contributes to increasing the potency of compounds, and the gray and red representations are carbons and oxygen atoms.

GCOD-5 (0,3,−3, any) have a positive coefficient, and so, improves the effectiveness of inhibitors (Figure 7). The GCOD (0,3,−3, any) is situated near the piperidine ring and has occupancy for a large majority of molecules, such as compounds **65** and **79**. The change of position of the piperidine ring in compounds **15**, **16**, and **30** is not favorable, so these do not have this GCOD.

**Figure 8:**
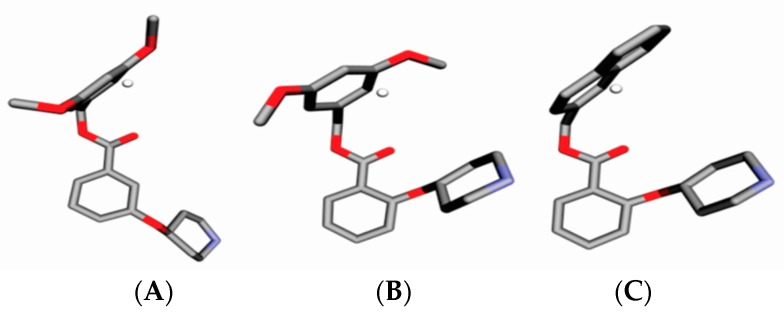
Representation of GCOD-6 (−1,−4,−3, any) (white sphere) and (**A**) compound **14**, (**B**) compound **19**, and (**C**) compound **22**. CODs occupancy represented by white spheres contributes to increasing the potency of compounds, and the gray and red representations are carbons and oxygen atoms.

GCOD-6 (−1,−4,−3, any) represents a non-specific IPE and also has a positive coefficient, indicating an increase in potency of compounds which have high occupation frequency for this descriptor (Figure 8). The molecules **14**, **19**, and **22** have a high occupation frequency for this GCOD, located near the benzene group. This grid cell suggests a hydrophobic region in the receptor close to the benzene ring, which should be making a π–π staking interaction between the aromatic ring of an inhibitor and one aromatic amino acid residue.

**Figure 9:**
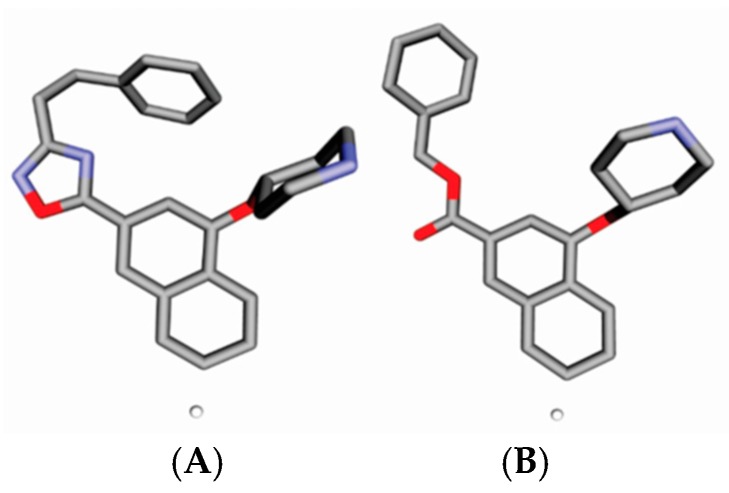
Representation of GCOD-7 (0,6,2, any) (white sphere) and (**A**) compound **62**, and (**B**) compound **59**, and the gray and red representations are carbons and oxygen atoms.

Lastly, GCOD-7 (0,6,2, any) (Figure 9) has a positive coefficient and shows a non-specific class. Molecules **59**–**63** have a high occupation frequency for this descriptor. The presence of naphthalene group in this grid cell increase the activity. In fact, it shows that the aromatic substituents in this position should be preferred.

In order to find new active structures, the information of the descriptors obtained by the Model B3 was used. Modifications to the structure of compounds **60**, **65**, and **81** are suggested and compounds **A**–**E** were proposed.

The proposed compounds **C**–**E**, exhibited predicted pIC_50_ higher than 81 (the best experimental compound). The structure of the five compounds and their predicted pIC_50_ values are shown in Table 3. The ADME (absorption, distribution, metabolism, and excretion) of a drug is an important property that can determine the utilization of molecules proposed in the therapeutic usage. For the evaluation of pharmacokinetic parameters for molecules **A**–**E** we used the Lipinski’s Rule of Five, where molecular properties are closely related to the oral bioavailability of a drug [20], wherein compounds should not violate more than one rule. In this rule, the compounds should present logP no more than 5, molecular weight of 500 Daltons, number of hydrogen bond acceptors (nON) of 10, number of hydrogen bond donors (nOHNH) of 5, and number of rotatable bonds (nrotb) no more than 10. The proposed molecules have been designed in the Molinspiration Online Property Calculation Software Toolkit [21] to evaluate the criteria discussed above.

The Molinspiration Online Property Calculation Software Toolkit [21] was used to analyze drug likeness (Lipinski’s Rule of Five) and the results are shown in Table 4. According to the data in Table 4, no compound violated the Lipinski’s Rule of Five.

## 4. Materials and Methods

### 4.1. Biological Data

In order to build QSAR models, 83 *Plasmodium falciparum* inhibitors were retrieved from Leatherbarrow et al. [15]. Twenty compounds (25%) were randomly selected to compose the test set (external validation). Two test groups were chosen. The first (Test Set I) has the following molecules: **1**, **3**, **5**, **6**, **12**, **16**, **20**, **30**, **33**, **39**, **40**, **50**, **56**, **57**, **61**, **65**, **66**, **69**, **76**, and **80**; Test Set II has the following molecules: **3**, **6**, **9**, **13**, **20**, **21**, **27**, **28**, **31**, **32**, **40**, **56**, **57**, **58**, **64**, **70**, **73**, **76**, **78**, and **82** (Table 5).

The biological activities of these compounds were reported as the negative logarithm of concentration capable of inhibiting 50% of the enzyme activity (IC_50_), measured using an adapted version of the sensitive fluorescence-based assay based on detection of CoA by 7-diethylamino-3-(4-maleimido-phenyl)-4-methylcoumarin [15]. Furthermore, in an effort to eliminate the potential noise that might have been introduced by the pooling of data sets from different sources, all pharmacological data are obtained from the same laboratory. The IC_50_ (µM) values were converted into molar units and then expressed in negative logarithmic units (pIC_50_), and are depicted in Table 4. The range of pIC_50_ values for the training and test set spans at least three orders of magnitude (4.00 to 7.30), and the biological activity values show a regular distribution over the whole range.

### 4.2. Molecular Dynamic Simulation (MDS)

The three-dimensional structure from 83 analogues (Table 5) were optimized in vacuum, without any restriction, and the partial atomic charges assigned using RM1 semiempirical Hamiltonian [22]. The MDS was carried out at 300 K, close to the temperature assays, with a simulation sampling time of 100 ps, and intervals of 0.001 ps. Thus, a total sample of 100,000 conformations of each compound was produced. MDS have been performed using the GROMACS 5.1 package [23].

### 4.3. Alignment Definition

**Figure 10:**
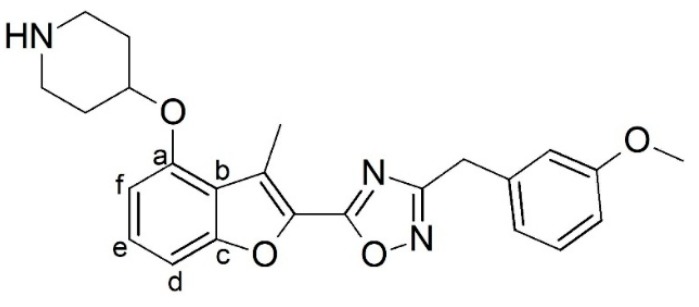
Ordered atom letter codes (a–f) used in the 4D-QSAR analysis defines the three trial alignments. Compound **81** (pIC_50_ = 7.301) is used to define the atom letter code.

As the compounds are structural analogs, we will assume that all molecules bind to the receptor in a similar mode. In general, the alignments are chosen to span the common framework of the molecules in the training and test sets [24,25,26]. In this work, ten alignments were performed using atoms of the (common) benzene ring. Three-ordered atom trial alignments were selected: (1) a-b-c, (2) a-b-d, (3) b-c-d, (4) c-d-f, (5) b-c-a, (6) b-a-c, (7) a-c-b, (8) c-b-a, (9) d-a-f, and (10) a-b-e (Figure 10). The order of the three ordered-atoms is important: the first atom specified for a molecule might be expected to occupy a similar location in space as the first atom specified for the second molecule. The conformational ensemble profile (CEP) for each compound obtained after the MDS step was overlaid onto a cubic lattice with grid cell size of 1A.

### 4.4. Interaction Pharmacophore Elements

According to the 4D-QSAR methodology, atoms of each compound are defined by seven types of interaction pharmacophore elements (IPEs). IPEs correspond to the interactions that may occur between ligand and the active site: (i) any type (any); (ii) nonpolar (np); (iii) polar-positive charge density (p+); 9iv) polar-negative charge density (p−); (v) hydrogen bond acceptor (hba); (vi) hydrogen bond donor (hbd); and (vii) aromatic systems (ar). The occupancy of the grid cells by each IPE type are recorded over the conformational assembly profile, and forms the set of grid cell occupancy descriptors (GCOD) to be utilized as the pool of trial descriptors in the model building and optimization process [16]. The idea underlying a 4D-QSAR analysis is that variations in biological responses were related to differences in the Boltzmann average spatial distribution of molecular shape with respect to the IPE. Thus, the normalized grid cell absolute occupancy, defined as the number of times that a cell was occupied by an atom type during the MDS, divided by the size of the CEP (1000 conformations), was used to define the GCODs.

4D-QSAR model calculation. In order to exclude data noise from databases generated by the alignments, partial least-squares (PLS) regression analysis was performed as a data reduction fit between the observed dependent variable measures and the corresponding set of GCOD values. Additionally, PLS identifies the most highly weighted GCODs from data set of local grid cells [16].

The two hundred GCODs with the highest weight from the data reduction were chosen to form the trial descriptor basis sets for model optimization by genetic function approximation (GFA) analysis [16]. Optimizations were initiated using 100 randomly generated models and 10,000–100,000 crossover operations. Mutation probability over the crossover optimization cycle was set at 10–30%. The smoothing factor, the variable that specifies the number of descriptors in the QSAR models, was varied between 1.0 and 3.0, in order to determine equations with no more than twelve terms. Each alignment was evaluated using the procedure described above.

The best models, resulting from the 4D-QSAR study were based on different criteria [18,26,27,28]:

(1) Coefficient of determination (r^2^): is a measure of how well the regression line represents the data.
(2) Adjusted cross-validated squared correlation coefficient (q^2^_adj_): allows the comparison between models with different number of variables.
(3) Correlation coefficient of external validation set (R^2^_pred_): reflects the degree of correlation between the observed (Y_Exp(test)_)and predicted (Y_Pred(test)_) activity data of the test set:
  (1) $$\;R_{\mathrm{Pred}}^{2}=1-\frac{\sum_{1}^{n}{\left(Y_{\mathrm{Exp}\left(\mathrm{test}\right)}-Y_{\mathrm{Pred}\left(\mathrm{test}\right)}\right)}^{2}}{\sum_{1}^{n}{\left(Y_{\mathrm{Exp}\left(\mathrm{test}\right)}-{\bar{Y}}_{\mathrm{Training}}\right)}^{2}}\;$$
  where ${\bar{Y}}_{\mathrm{Training}}$ is average value for the dependent variable for the training set.
(4) Modified r^2^ (r^2^_m(test)_) equation determining the proximity between the observed and predicted values with the zero axis intersection:
  (2) $$\;r_{m\left(\mathrm{test}\right)}^{2}=r^{2}(1-|\sqrt{r^{2}-r_{0}^{2}}|)\;$$
(5) Y-randomization (R^2^r) consists of the random exchange of the independent variable values. Thus, the R^2^r value must be less than the correlation coefficient of the non-randomized models.
(6) R^2^p penalizes the model R^2^ for the difference between the squared mean correlation coefficient (R^2^_r_) of randomized models and the square correlation coefficient (r^2^) of the non-randomized model:
  (3) $$\;R_{p}^{2}=r^{2}*\sqrt{r^{2}-R_{r}^{2}}\;$$

### 4.5. Conformational Selection

In the 4D-QSAR method, the conformation of each compound can be postulated as the lowest-energy conformer state from the set sampled for each compound, which predicted the maximum activity using the optimum 4D-QSAR model [16,29,30,31,32].

## 5. Conclusions

In summary, 4D-QSAR models for NMT inhibitors were built and evaluated. Two test groups were evaluated for the ten tested alignments. The best model was obtained from Alignment B3, and generated an equation with seven descriptors, six of which have positive coefficients and one a negative coefficient. Model B3 showed a satisfactory statistical quality and predictive abilities as shown by r^2^ = 0.757, q^2^ = 0.702, q^2^_adjusted_ = 0.634, R^2^_pred_ = 0.746, R^2^_m_ = 0.716, and R^2^_p_ = 0.609. Furthermore, it showed low values of R^2^_r_ = 0.110, and ∆R^2^_m(test)_ = 0.133. 4D-QSAR analysis indicated an important role of acceptor hydrogen bonding groups and aromatic groups, allowing to propose five structures. These have proved more active than compound **81**, in addition to being assessed by the Lipinski’s Rule. Accordingly, these molecules may be considered promising prototypes against malaria.

## Figures and Tables

**Table 1:** Statistical parameters evaluated in the 4D-QSAR analysis for the ten performed alignments of Test I.

Alignment	r^2^	RMSE_C_	q^2^_adj_	RMSE_CV_	R^2^_Pred_	RMSE_P_	r^2^_m_	R^2^_r_	R^2^_p_
A1	0.746	0.481	0.607	0.549	0.532	0.65	0.71	0.312	0.82
A2	0.744	0.478	0.608	0.548	0.548	0.663	0.692	0.343	0.799
A3	0.761	0.469	0.609	0.546	0.508	0.702	0.735	0.182	0.994
A4	0.708	0.508	0.576	0.579	0.588	0.595	0.645	0.287	0.825
A5	0.736	0.511	0.589	0.566	0.477	0.698	0.766	0.245	0.895
A6	0.739	0.477	0.582	0.563	0.567	0.637	0.67	0.286	0.83
A7	0.722	0.503	0.584	0.571	0.555	0.656	0.683	0.291	0.831
A8	0.746	0.445	0.605	0.551	0.62	0.59	0.606	0.216	0.891
A9	0.734	0.491	0.578	0.566	0.547	0.684	0.693	0.25	0.861
A10	0.723	0.519	0.583	0.572	0.503	0.676	0.74	0.311	0.816

r^2^: Coefficient of determination; RMSE_c_: root mean square deviation of calibration; q^2^_adj_: adjusted cross-validated squared correlation coefficient; RMSE_cv_: root mean square deviation of cross validation; R^2^_pred_: correlation coefficient of external validation; RMSEp: root mean square deviation of prediction; r^2^_m_(test): Equation (3) (Materials and Methods); R^2^_r_: Y-randomization; R^2^_p_: Equation (2) (Materials and Methods).

**Table 2:** Statistical parameters evaluated in the 4D-QSAR analysis for the ten performed alignments of Test II.

Alignment	r^2^	RMSE_C_	q^2^_adj_	RMSE_CV_	R^2^_Pred_	RMSE_P_	R^2^_m_	R^2^_r_	R^2^_p_
B1	0.728	0.504	0.617	0.544	0.728	0.532	0.688	0.301	0.476
B2	0.728	0.515	0.607	0.553	0.763	0.496	0.749	0.289	0.482
B3	0.757	0.472	0.634	0.527	0.746	0.515	0.716	0.11	0.609
B4	0.704	0.549	0.585	0.573	0.782	0.476	0.765	0.253	0.473
B5	0.725	0.5	0.601	0.55	0.706	0.553	0.692	0.198	0.526
B6	0.692	0.559	0.576	0.581	0.771	0.489	0.755	0.272	0.448
B7	0.69	0.556	0.581	0.577	0.751	0.509	0.735	0.289	0.437
B8	0.73	0.514	0.6	0.55	0.77	0.489	0.75	0.209	0.527
B9	0.723	0.528	0.605	0.555	0.786	0.472	0.773	0.229	0.508
B10	0.744	0.501	0.619	0.542	0.779	0.48	0.744	0.289	0.502

r^2^: coefficient of determination; RMSE_c_: root mean square deviation of calibration; q^2^_adj_: adjusted cross-validated squared correlation coefficient; RMSE_cv_: root mean square deviation of cross validation; R^2^_pred_: correlation coefficient of external validation; RMSEp: root mean square deviation of prediction; r^2^_m_(test): Equation (3) (Materials and Methods); R^2^_r_: Y-randomization; R^2^_p_: Equation (2) (Materials and Methods).

**Table 3:** Structures of the compounds proposed and the predicted pIC_50_ values based on Model B3.

No.	Structure	pIC_50_	No.	Structure	pIC_50_
**A**	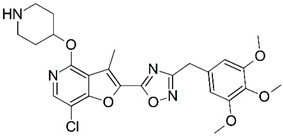	7.014	**B**	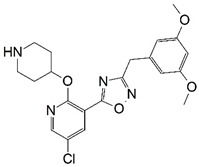	7.171
**C**	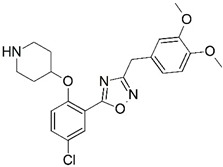	7.622	**D**	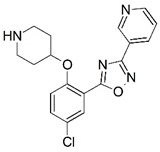	8.161
**E**	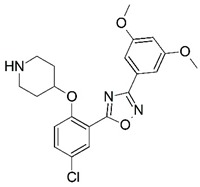	7.894			

**Table 4:** Calculated parameters of the Lipinski rule of five for the proposed molecules.

Molecule	miLogP	MW	*n*_ON_	*n*_OHNH_	*n*	*n*_violations_
A	3.13	514.97	10	1	8	1
B	2.74	430.89	8	1	7	0
C	3.13	415.88	7	1	6	0
D	2.41	356.81	6	1	4	0
E	3.52	415.88	7	1	6	0

**Table 5:** Chemical structures and experimental pIC_50Exp_ (M) values of *Plasmodium falciparum* inhibitors. Test Set I compound numbers are marked with an asterisk. Test Set II compound numbers are underlined.

No.	Structure	pIC_50_	No.	Structure	pIC_50_
1 *	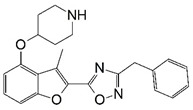	6.155	**2**	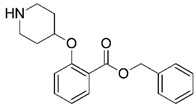	4.000
3 *	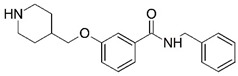	4.000	**4**	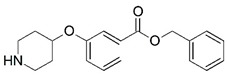	4.000
5 *	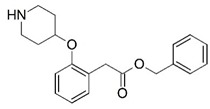	4.000	6 *	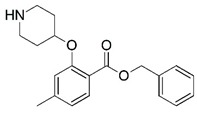	4.000
**7**	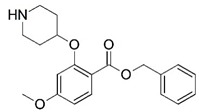	4.000	**8**	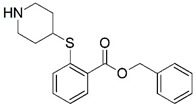	4.000
**9**	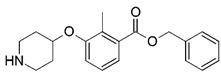	4.000	**10**	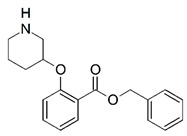	4.000
**11**	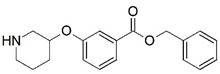	4.000	12 *	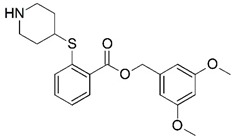	5.721
**13**	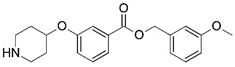	4.785	**14**	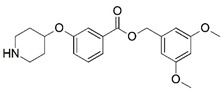	5.113
**15**	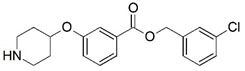	4.000	16 *	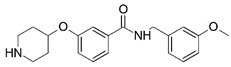	4.000
**17**	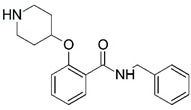	4.000	**18**	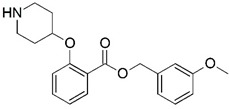	4.745
**19**	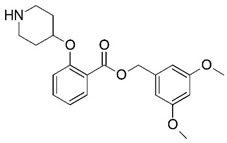	5.215	20 *	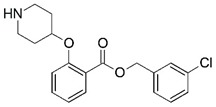	4.366
**21**	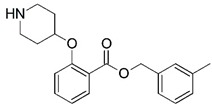	4.000	**22**	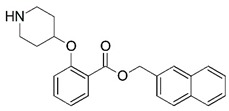	4.000
**23**	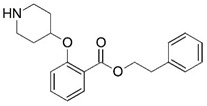	4.000	**24**	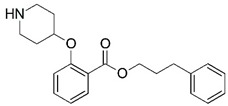	4.000
**25**	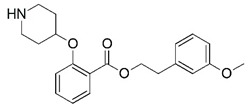	4.000	**26**	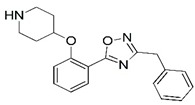	5.699
**27**	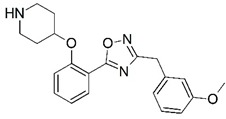	6.400	**28**	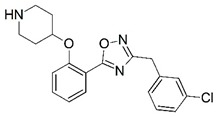	6.102
**29**	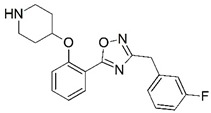	5.780	30 *	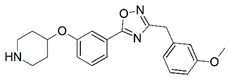	6.398
**31**	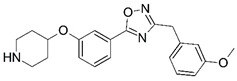	5.796	**32**	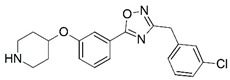	5.420
33 *	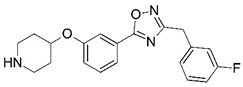	5.292	**34**	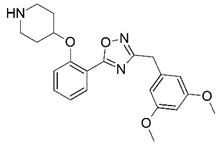	6.456
**35**	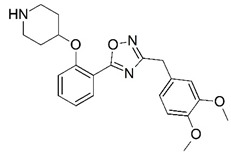	6.678	**36**	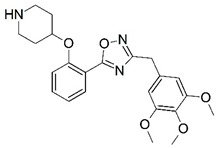	6.468
**37**	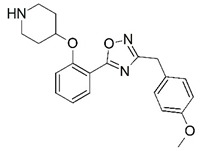	5.131	**38**	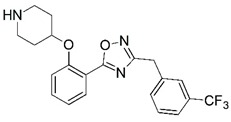	5.585
39 *	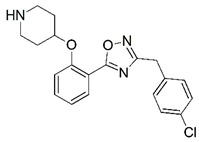	4.730	40 *	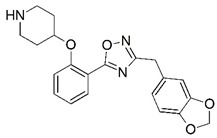	5.585
**41**	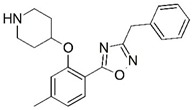	5.886	**42**	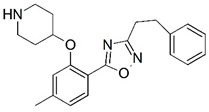	5.284
**43**	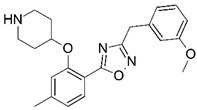	6.000	**44**	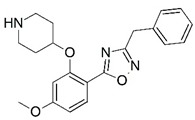	5.602
**45**	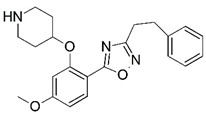	4.876	**46**	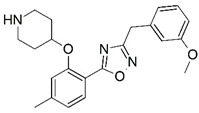	6.319
**47**	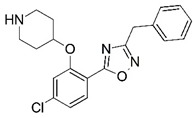	6.215	**48**	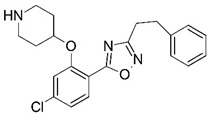	6.051
**49**	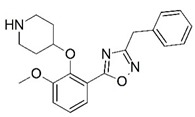	4.445	50 *	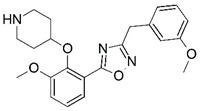	4.958
**51**	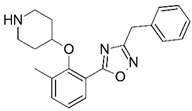	4.086	**52**	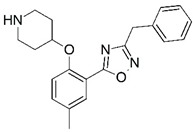	4.217
**53**	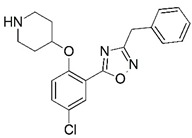	6.398	**54**	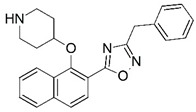	5.569
**55**	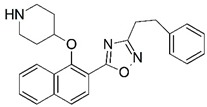	4.663	56 *	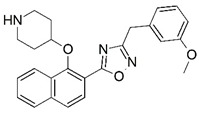	6.229
57 *	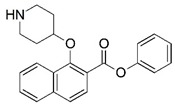	4.182	**58**	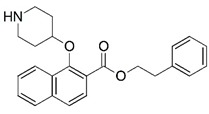	5.056
**59**	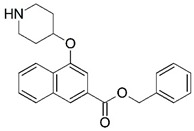	5.009	**60**	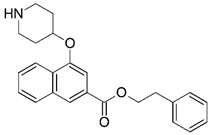	5.149
61 *	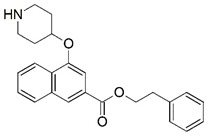	5.886	**62**	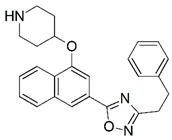	5.886
**63**	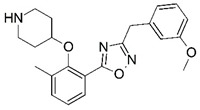	4.801	**64**	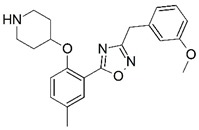	5.201
65 *	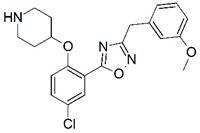	6.959	66 *	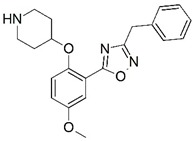	5.538
**67**	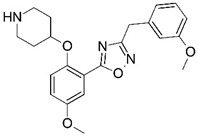	6.482	**68**	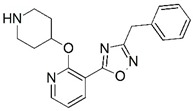	5.921
69*	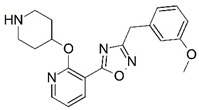	6.769	**70**	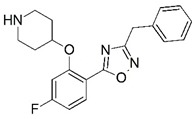	5.569
**71**	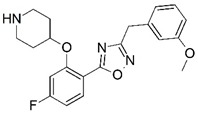	6.824	**72**	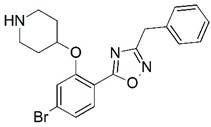	6.051
**73**	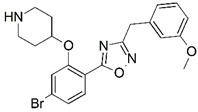	7.222	**74**	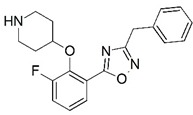	6.000
**75**	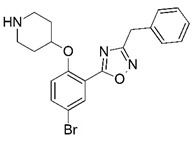	6.620	76 *	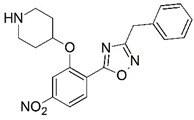	6.638
**77**	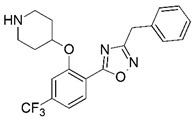	5.495	**78**	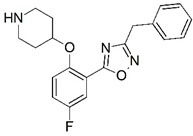	5.959
**79**	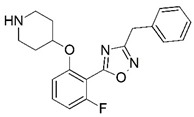	6.181	80 *	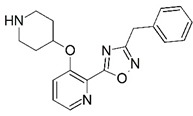	5.187
**81**	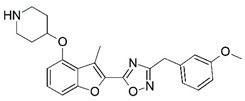	7.301	**82**	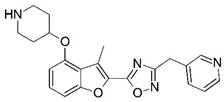	6.921
**83**	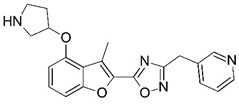	6.201			

* Test set group of compounds.
